# Supplementary material for: The TP53 Codon 72 Arginine Polymorphism Is Found with Increased TP53 Somatic Mutations in HPV(−) and in an Increased Percentage among HPV(+) Norwegian HNSCC Patients
Source: Biomedicines. 2023 Jun 26;11(7):1838. doi: 10.3390/biomedicines11071838 (PMC10376802; doi:10.3390/biomedicines11071838)
Supplement: Supplementary file 1 [file biomedicines-11-01838-s001.zip › biomedicines-2436943-supplementary.pdf]

Supplementary Table S1 Exact *TP53* mutations detected by HPV status of patients

Predicted protein change	Legacy Identifier (COSMIC)	Mutation type	FATHMM-MKL prediction score	No. HPV(+) tumors	No. HPV(-) tumors
P36S	2745156	Missense	0.04	1	0
W91*	44192	Nonsense	0,97	0	1
Y103*	45307	Nonsense	0,98	0	1
F113del	45143	Deletion - In frame	n.a.	0	1
L137Q	44745	Missense	1,00	0	1
A161S	43549	Missense	0,95	0	1
A161T	10739	Missense	0.94	0	1
I162_Q165del	n.a.	Deletion -In frame	n.a.	1	0
Y163*	43820	Nonsense	0,96	0	1
S166*	44467	Nonsense	0,98	0	1
V173L	43559	Missense	0,99	0	1
V173E	45731	Missense	0,99	0	2
C176F	10645	Missense	0,99	0	1
H178_H179del	n.a.	Deletion -In frame	n.a	0	1
H179L	43635	Missense	0,99	0	1
H179R	10889	Missense	0,99	0	1
H193L	11066	Missense	0.99	0	2
H193R	10742	Missense	0.99	1	1
R196*	10705	Nonsense	0,96	0	3
E204*	10804	Nonsense	0,48	0	1
E204Gfs*43	n.a.	Deletion -Frameshift	n.a.	0	1
Y205D	43844	Missense	0,99	0	1
T211I	43939	Missense	0,99	0	1
H214R	43687	Missense	0,99	0	1
Y220C	10758	Missense	0.99	0	2
E224=	44754	Coding silent	0.99	0	1
C238Y	11059	Missense	0.99	0	2
G244C	11524	Missense	0,99	0	1
R248W	10656	Missense	0.94	0	2
R248Q	10662	Missense	0.98	0	1

T256I	45973	Missense	0,99	0	1
E258G	44168	Missense	0,99	0	1
V272L	10859	Missense	0.99	0	1
R273H	10660	Missense	1.00	0	2
C275Y	10893	Missense	1.00	0	1
D281N	43596	Missense	1.00	0	1
R282W	10704	Missense	0.99	0	2
E286*	43919	Nonsense	1,00	0	1
E286G	43565	Missense	0,99	0	1
R290H	44017	Missense	0.31	0	1
S303R	n.a	Missense	0,64	0	1
S303Kfs*39	n.a.	Deletion -Frameshift	n.a.	0	1
K320*	44335	Nonsense	0,99	0	1
E326*	11570	Nonsense	1,00	0	1
T329Hfs*8	100040	Insertion -Frameshift	n.a.	0	1

COSMIC: Catalogue Of Somatic Mutations In Cancer (https://cancer.sanger.ac.uk)

FATHMM: Functional Analysis through Hidden Markov Models

HPV: Human papilloma virus

n.a.: not applicable
